# Case of Vitiligooidea, with Remarks

**Published:** 1853-04

**Authors:** W. H. Ranking

**Affiliations:** M. D. (Cantab.) Norwich


*Case of Vitiligooidea, with remarks.* By W. H. Ranking, M. D. (Cantab.) Norwich.—In the summer of 1850, the following case presented itself to my notice, and a very brief inspection was sufficient to lead me to believe that I had encountered a disease, of which no distinct account had been given by writers on dermatology. I took notice of the case at the time, with reference to publication, but other occupations interfered with my intention, and the circumstance had all but escaped my recollection, when I met with the description of this disease, by Dr. Gull, under the term *vitiligooidea,* the identity of which with my own case could not fail to be recognized. The instances of this curious malady hitherto put on record do not amount to more than five. I have accordingly endeavored to give a succinct account of what is known thereupon.

My patient, Mary B——, a married woman, aged twenty-nine, consulted me, in June, 1850, for obstinate and severe jaundice. Inquiry into her history elicited the facts that her health had been good until three years previously, when, after an attack of dyspepsia, jaundice supervened, and had continued till the time of her visit to me, uninfluenced by treatment, including several salivations. At this time she was universally and deeply jaundiced; but the appearance which immediately and more strongly attracted my attention, was a peculiar deposit on the skin surrounding the eyes, and which, on further investigation, I found to be abundantly distributed over other portions of the body. She informed me that about twelve months ago, spots of this peculiar deposit first appeared on the shoulders, and have since shown themselves on the face, arms, hands and lumbar regions. In the face, it has assumed a symmetrical disposition, extending along each eyelid, and down the side of the nostrils. On the shoulder the spots are circular and distinctly elevated. Along the inside of the elbows, and on the hands, the deposit follows the flexures of the joints, being flat and linear on the palmar aspect, and more tubercular and rounded on the dorsal. The color of this deposit is of a whitish-yellow, resembling more nearly than anything else the atheromatous patches so commonly found in the aorta. On the face and palms of the hands it is but little elevated; and, as in atheroma, appears to be deposited immediately under the epithelium. On the shoulder and in the dorsal region the spots are circular and prominent, bearing no inconsiderable resemblance to split peas.

At the time of my seeing this case the peculiar affection was still on the increase, fresh spots showing themselves almost daily. They were tender to the touch, and were the seat of a burning sensation, which prevented the patient using her hands without acute suffering.

The history of the jaundice pointed to the conclusion that it depended upon permanent occlusion of the common duct. The woman died soon after my seeing her, under the care of another practitioner, and, as far as I could learn, from severe and rapid peritonitis.

The only references to this singular cutaneous disease which I have been able to meet with are contained in a paper published in "Guy's Hospital Reports;" for although Willan alludes to a rare disease under the term *vitiligo*, which has some points of resemblance, the full comparison of the two is unfavorable to the notion of their identity. The term used by Willan has, however, led Dr. Gull to give to the disease in question the name *vitiligooidea.* The cases narrated by him are four in number. The first is that of a married woman, aged forty-two, who had been deeply jaundiced for two years. At the end of fourteen months this pecnliar change in the integument began to show itself on the eyelids, assuming a perfect symmetrical form, as well as in the palms of the hands, where, as in my case, it followed accurately the flexures of the joints. The disease remained stationary till her death.

A second patient was admitted into Guy's Hospital, laboring under diabetes; but in this instance the eruption was so far different in its aspect that I cannot think Dr. Gull justified in associating it with the former case.

The third instance is that of a married woman, like the first, the subject of jaundice. The skin diseases commenced at the end of fourteen months, appearing first on the hands and subsequently affecting the eyelids. This case is now, I believe, under treatment, but the disease shows a tendency to increase rather than diminish.

The fourth case described by Dr. Gull is, in every respect, similar, both as to the prior existence of jaundice, and the distribution of the cutaneous deposit.

I have ventured to call attention to the above singular disease, chiefly on account of its singularity, and not with the expectation of being able to offer any elucidation of its pathology or treatment. To speculate on the former might, until further experience is afforded, be an unprofitable exercise; nevertheless, it is impossible to overlook the important fact of some connextion between the cutaneous deposit and jaundice of an aggravated degree and prolonged duration. In every case reported, obstinate and severe jaundice had existed for several months prior to the appearance of the skin malady. Can it be that, like the cretaceous deposits of gout, this was an attempt at elimination of noxious matter necessarily contained in blood, in which was suspended the elements of bile? I regret much that I had no opportunity of instituting a microscopic examination of the deposit; but its *prima facie* resemblance to atheroma would warrant the idea that, like the latter exudation, the peculiar deposit in question contained cholesterine, a principle which is well known to enter largely into the composition of the biliary secretion.

This explanation of the composition and the pathological significance of the vitiligoid deposit is, as we have premised, simple hypothesis; but, such as it is, I would offer it, in order to elicit observations.

Respecting treatment, nothing can be said. If, as I imagine, the affection is but a symptom of prolonged retention of bile, or its elements in the blood, nothing in the way of amendment can be anticipated, unless the original malady be removed, when possibly the absorbents might spontaneously remove the deposit. In the cases hitherto reported no decided amendments followed any of the means adopted.—*London Lancet*, Feb., 1853.

